# Supplementary material for: Hybrid Models and Biological Model Reduction with PyDSTool
Source: PLoS Comput Biol. 2012 Aug 9;8(8):e1002628. doi: 10.1371/journal.pcbi.1002628 (PMC3415397; doi:10.1371/journal.pcbi.1002628)
Supplement: Text S4 — Complete source code for the PyDSTool package (version 0.88.120504). Includes API documentation and help files linking to web pages. This file is identical to the current public release on Sourceforge.net. (ZIP) [file pcbi.1002628.s004.zip › PyDSTool/html/PyDSTool.Toolbox.neuro_data-module.html]

xml version="1.0" encoding="ascii"?


PyDSTool.Toolbox.neuro\_data


| Home | Trees | Indices | Help | PyDSTool |
| --- | --- | --- | --- | --- |

Package PyDSTool ::
Package Toolbox ::
Module neuro\_data

[hide private]

[frames] | no frames]

# Module neuro\_data

source code


|  |  |
| --- | --- |
| Classes | [hide private] |

get\_spike\_model  
Qualitative test for presence of spike in model trajectory data
using events to identify spike times.

get\_spike\_data  
Qualitative test for presence of spike in noisy data.

get\_burst\_duration

get\_burst\_active\_phase

get\_burst\_dc\_offset

get\_burst\_passive\_extent

burst\_feature  
Embed the following sub-features, if desired: get\_burst\_X, where X
is a number of feature types defined in this module.

get\_burst\_spikes  
Requires a get\_spike\_data and get\_spike\_model instance to be the
only sub-features (supplied as a dict with keys 'is\_spike\_data' and
'is\_spike\_model').

get\_burst\_peak\_env  
Requires tol and num\_samples parameters.

get\_burst\_trough\_env  
Requires tol and num\_samples parameters.

get\_burst\_isi\_env  
Requires tol and num\_samples parameters.

get\_burst\_upsweep

get\_burst\_downsweep

get\_burst\_num\_spikes

get\_burst\_period\_info

spike\_metric  
Measures the distance between spike time and height, using an
inherent weighting of height suited to neural voltage signals (0.05
of time distance).

spike\_feature  
pars keys: tol

geom\_feature  
Measures the residual between two 1D parameterized geometric curves
(given as Trajectory objects).

estimate\_spiking  
Estimate pattern of spiking in tonic or burst patterns.

spike\_envelope  
Find an amplitude envelope over a smooth 1D signal that features
roughly periodic spikes.


|  |  |
| --- | --- |
| Functions | [hide private] |

|  |  |
| --- | --- |
| find\_internal\_extrema(pts, noise\_tol=0)  Find an interior (local) maximum and minimum values of a 1D pointset, away from the endpoints. | source code |


|  |  |
| --- | --- |
| Function Details | [hide private] |

|  |  |
| --- | --- |
| find\_internal\_extrema(pts, noise\_tol=0) | source code |

Find an interior (local) maximum and minimum values of a 1D pointset,
away from the endpoints. Returns a dictionary mapping 'local\_max' ->
(index\_max, xmax), 'local\_min' -> (index\_min, xmin), whose values are
None if the pointset is monotonic or is close enough so that the global
extrema are at the endpoints.

Use noise\_tol > 0 to avoid getting a local extremum right next to
an endpoint because of noise.

Also returned in the dictionary for reference: 'first' -> (0,
<start\_endpoint\_value>), 'last' -> (last\_index,
<last\_endpoint\_value>), 'global\_max' -> (index, value),
'global\_min' -> (index, value)

Assumes there is only one interior (max, min) pair in pts, otherwise
will return an arbitrary choice from multiple maxima and minima.

  


| Home | Trees | Indices | Help | PyDSTool |
| --- | --- | --- | --- | --- |

|  |  |
| --- | --- |
| Generated by Epydoc 3.0.1 on Fri May 4 15:24:05 2012 | http://epydoc.sourceforge.net |
